# Modification of a Rodent Hindlimb Model of Secondary Lymphedema: Surgical Radicality versus Radiotherapeutic Ablation

**DOI:** 10.1155/2013/208912

**Published:** 2013-11-14

**Authors:** Hyung Sub Park, In Mok Jung, Geum Hee Choi, Soli Hahn, Young Sun Yoo, Taeseung Lee

**Affiliations:** ^1^Department of Surgery, Seoul National University College of Medicine, Seoul 110-799, Republic of Korea; ^2^Department of Surgery, Seoul National University Bundang Hospital, 166 Gumi-ro, Bundang-gu, Seongnam-si, Gyeonggi-do 463-707, Republic of Korea; ^3^Department of Surgery, Seoul Metropolitan Government Seoul National University Boramae Medical Center, Seoul 156-707, Republic of Korea; ^4^Department of Surgery, Chosun University Hospital, Chosun University College of Medicine, Gwangju 501-717, Republic of Korea

## Abstract

Secondary lymphedema is an intractable disease mainly caused by damage of the lymphatic system during surgery, yet studies are limited by the lack of suitable animal models. The purpose of this study was to create an improved model of secondary lymphedema in the hindlimbs of rodents with sustained effects and able to mimic human lymphedema. This was achieved by combining previously reported surgical methods and radiation to induce chronic lymphedema. Despite more radical surgical destruction of superficial and deep lymphatic vessels, surgery alone was not enough to sustain increased hindlimb volume. Radiotherapy was necessary to prolong these effects, with decreased lymphatic flow on lymphoscintigraphy, but hindlimb necrosis occurred after 4 weeks due to radiation toxicity. The applicability of this model for studies of therapeutic lymphangiogenesis was subsequently tested by injecting muscle-derived stem cells previously cocultured with the supernatant of human lymphatic endothelial cells in vitro. There was a tendency for increased lymphatic flow which significantly increased lymphatic vessel formation after cell injection, but attenuation of hindlimb volume was not observed. These results suggest that further refinement of the rodent hindlimb model is needed by titration of adequate radiation dosage, while stem cell lymphangiogenesis seems to be a promising approach.

## 1. Introduction

Lymphedema is defined as the accumulation of tissue fluid as a consequence of impaired lymphatic drainage [1]. Its etiology may be either congenital or acquired, and it is estimated to affect approximately 140 to 250 million people worldwide [2]. The acquired form of lymphedema is more common and is mainly caused by destruction of the lymphatic system during surgery or radiation therapy. Unfortunately there is no known definite cure for lymphedema and most of the current treatment strategies are focused on conservative measures with treatment of secondary complications such as cellulitis/lymphangitis or malignant tumors.

Our understanding about the pathologic mechanisms of this disease and therapeutic approaches is limited by the lack of suitable animal models. The importance of animal models for preclinical studies is indisputable, and many researchers have proposed several animal models in the last several decades, which include canine, rabbit, and rodent models, but none of them have been able to reliably reproduce a sustained chronic effect similar to that found in human secondary (postsurgical) lymphedema [3]. The first models were created in dogs as early as 1888 by ligation of the lymphatic trunks in the hindlimb [4], and many modifications have been made since then, but none of the methods gained great acceptance because of the complexity of the procedure and the long latency before the appearance of chronic lymphedema [5]. An alternative model was the rabbit ear model, first proposed in 1977, which had the anatomic advantage of having superficially located lymphatics and nonexistent deep lymphatic systems, making model creation easier and more reliable [6]. However, this method had problems regarding difficult reproducibility by other researchers with high dependence on the animals and conditions used. The rodent model was first introduced in the 1980s and is most widely used nowadays because of its easy accessibility, low cost, and shorter duration to obtain clinically relevant observations. Additionally, the rodent lymphatics present muscular patterns that are similar to humans than any other species, making physiologic studies more feasible [5]. There are two models of lymphedema in rodents: the tail model and the hindlimb model. The tail model is being used in most recent studies because of its procedural simplicity and its effectiveness in providing information about the molecular aspects of lymphangiogenesis. The hindlimb model, on the other hand, is more complex to create but is physiologically and anatomically more similar to human postsurgical lymphedema than the tail model and has the advantage of providing abundant tissue for harvesting.

These animal models can be used for studies of therapeutic lymphangiogenesis, which involves the regeneration of lymphatic vessels to restore lymphatic flow and improve lymphedema. Lymphangiogenesis has been shown to occur in adult tissues during inflammation, wound healing, and tumor metastasis [7]. Research in lymphangiogenesis has been possible due to identification of regulatory molecules and markers specific to the lymphatic endothelium, which include Prox-1, lymphatic vessel hyaluronan receptor-1 (LYVE-1), vascular endothelial growth factor receptor-3 (VEGFR-3 or Flt-4), and podoplanin. These markers are now specifically used as markers of lymphatic vessels.

In this study, we set out to determine whether a sustainable model of lymphedema can be created by more radical destruction of the lymphatic vessels using surgical and radiotherapeutic methods in rodent hindlimbs. The applicability of this model for studies of therapeutic lymphangiogenesis was investigated by injecting lymphatic endothelial cell (LEC) precursors previously differentiated from muscle-derived stem cells in vitro into the hindlimbs, and the potential role of stem cell therapy was assessed.

## 2. Materials and Methods

### 2.1. Animal Model for Lymphedema and Study Design

**Figure 1:**
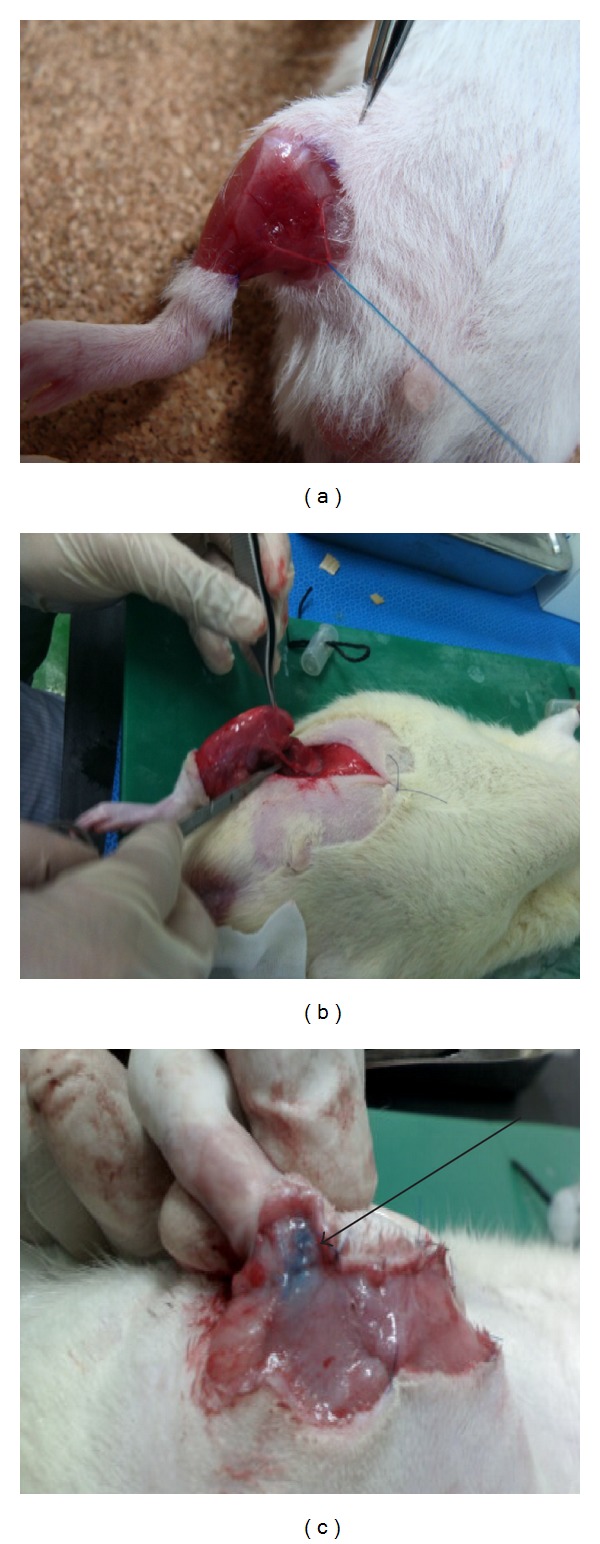
Formation of lymphedema in the right hindlimb of Balb/c mice. (a) Meticulous dissection and preservation of the neurovascular bundle by gentle retraction. (b) Circumferential resection of strip of skin, subcutaneous tissue, and muscle to obliterate deep lymphatics. (c) Staining of lymph nodes and lymphatics after injection of methylene blue (black arrow) for lymphatic obliteration and lymph node dissection.

Under the approval of the Institutional Animal Care and Use Committee (IACUC) of Seoul National University Bundang Hospital for the whole study, Balb/c mice (male, 4 weeks old) were anesthetized using an intraperitoneal injection of 0.2 mL tiletamine/zolazepam (Zoletil, Virbac) and 0.15 mL xylazine (Rompun, Bayer). For detection of the lymphatic system, 0.05 mL of methylene blue was injected subcutaneously in the right distal hindlimb and the limb was intermittently flexed and extended for 30 min. A circumferential incision was made on the right hindlimb and a 1 cm wide circumferential strip of skin and subcutaneous tissue was resected. During the procedure, the medial neurovascular bundle was saved by meticulous dissection and gentle retraction to separate it from the surrounding tissue (Figure 1(a)). Further resection of the underlying muscle was done to destroy the deep lymphatic system (Figure 1(b)), and electrocauterization of the stained lymphatics as well as circumferentially along the proximal and distal margins was performed. The inguinal lymph nodes were also removed surgically using a surgical microscope system (Carl Zeiss) and remnant lymphatics were electrocauterized (Figure 1(c)). The resected muscle was restored by fixation to the surrounding tissue using a 5-0 polyglactin suture and the wound was covered with a strip of surgical glove to protect the wound from external damage or dehydration.

A total of 24 mice underwent this procedure for creation of lymphedema and were divided into 3 groups. In the Surgery group (*n* = 8), the above described surgical procedure was performed only, while, in the Surgery+RT group (*n* = 8), mice were exposed to 4500 cGy/3 fractions of radiotherapy at day 5 after surgery. Fractional doses of 1500 cGy were given to the inguinal area for 3 consecutive times with intervals between each fraction using a 6 MeV electron (Varian 21 EX linear accelerator), while the rest of the mouse body (except for the inguinal area) was shielded from radiation using lead blocks. In a third group, named the Cell therapy group (*n* = 8), the same surgical and RT procedures were performed, and injection of 1 × 10^7^ cells of LEC precursors obtained from in vitro MDSC cocultured with HLEC (50% sup.) was given at 3 different locations in the hindlimb at day 5 immediately after RT. The same volume of saline was injected in the hindlimbs (both operated and contralateral nonoperated limbs) of the other groups to correct for volume discrepancies during subsequent volumetric analyses.

### 2.2. Water Displacement Volumetric Analysis

The degree of lymphedema was quantified by measuring the volume of the right (operated) hindlimb using a water displacement volumetry method. A 1.8 mL polypropylene tube (Nalge Nunc) was fully filled with saline and the right hindlimb was inserted into the tube up to the most proximal margin of the circumferentially excised wound. The overflowed saline was refilled using a 1 mL syringe and the volume of saline needed to fully refill the tube was indirectly used as an indicator of hindlimb volume. All mice were anesthetized during this procedure and the contralateral left limb was used as a control for normal hindlimb volume. Measurement was performed at 1, 3, and 5 days and 1, 2, 4, and 8 weeks and each measurement was performed 3 times by a single researcher to obtain a mean value. 

### 2.3. Lymphoscintigraphy

Lymphoscintigraphy was performed at 8 weeks using a NanoSPECT/CT device (Bioscan). After intraperitoneal anesthesia, technetium-99m antimony sulfur colloid (Tc-99m ASC) was injected subcutaneously in the right foot and frames were taken at a speed of 15 seconds per frame for the first 5 minutes. A delayed frame was taken 40 minutes after injection. All necessary safety measures regarding radioactivity issues were taken. 

### 2.4. LYVE-1 IHC Staining of Lymphatic Capillaries

Tissue specimens from the right hindlimb were obtained at 1, 2, 4, and 8 weeks for immunohistochemical (IHC) staining and RT-PCR of lymphatic markers. For LYVE-1 3,3′-diaminobenzidine (DAB) IHC, tissues were fixed in 4% formalin, embedded in paraffin, and sectioned into 5–7 *μ*m slices. After deparaffinization, rehydration, and blocking, the tissues were incubated with primary antibody for LYVE-1 (1 : 100, Abcam) at 4°C overnight. The next day, DAB staining was performed with the REAL Envision Detection System, Peroxidase/DAB+, and Rabbit/Mouse (Dako) according to the manufacturer's instructions, which sequentially conjugates secondary goat antigen and stains for DAB. LYVE-1 positive tubular structures were counted by from 4 random high-power microscope fields with Image Pro Plus 4.5 (Cybernetics Inc.) and the mean value was calculated. 

### 2.5. In Vitro Lymphatic Differentiation of MDSCs and Lymphatic Marker Expression

The injected cells in the Cell therapy group were obtained by isolation of MDSCs from the gastrocnemius muscle of 4–6-week-old Balb/c mice using a modified preplate technique, as previously described [8, 9]. Human lymphatic endothelial cells (HLEC, ScienCell Research Laboratories) were cultured for 3 days until 80% confluence was achieved and the supernatant was moved to a new tube. Isolated MDSCs (50%) and the supernatant of HLECs (50%) were placed in a new culture medium and cocultured for 4 weeks for lymphatic differentiation.

To determine the differentiation of MDSCs to LEC precursors, the lymphangiogenic characteristics of MDSCs cocultured with 50% supernatant of HLECs were compared with native MDSCs and HLECs. The respective cells were seeded on plate slides (Nalge Nunc), fixed with cold methanol, and blocked for 30 min with 5% blocking solution (Dako). The primary antibody Prox-1 (1 : 100, Abcam) was added and incubated overnight at 4°C. For DAB staining, the REAL Envision Detection System, Peroxidase/DAB+, and Rabbit/Mouse (Dako) were used as described previously. Counterstaining with hematoxylin and mounting with DAPI were then performed and the slides were observed under microscopy. For immunofluorescence (IF) staining, the respective fluorescein isothiocyanate-(FITC-labeled) secondary antibody was incubated for 3 h at 4°C and mounted with DAPI and the slides were observed under microscopy. The expression of Prox-1 was quantified for the three different cell types by counting the number of positive cells from 4 random high-power fields, and the relative expression rate of native MDSCs and cocultured MDSCs against HLECs was determined.

Reverse transcriptase polymerase chain reaction (RT-PCR) was performed for lymphatic markers Prox-1, Flt-4, and podoplanin. Total RNA from the respective cells were extracted using the RNeasy Mini Kit (Qiagen) according to the manufacturer's instructions, and the quantity and quality of the extracted RNA were determined by absorbance at 260 nm and 280 nm wavelengths using the SmartSpec Plus Spectrophotometer (Bio-Rad). An oligonucleotide deoxythymidine primer was added to 2 *μ*g of the extracted total RNA and incubated at 65°C for 10 min. The denatured RNA was mixed with 4 *μ*L of 5x reaction buffer, 1 mM dNTP, 20 U of RNase inhibitor, and reverse transcriptase BioScript (Bioline) to make a final volume of 20 *μ*LcDNA. For PCR amplification, cDNA equivalent to 500* *ng of starting RNA was mixed with 20 pmol of forward and reverse primers with 10x PCR buffer (MangoMix, Bioline). The sequences of the PCR primers for lymphatic markers are shown in Table 1. PCR amplification was performed with PTC-200 PCR Thermal Cycler (MJ Research) using the following cycling conditions: 94°C for 2 min (initial denaturation), 94°C for 30 s, 53–55°C for 45 s, 72°C for 30 s (35 cycles), and 72°C for 10 min (final extension). PCR products were subjected to electrophoresis on 1-2% agarose gels containing ethidium bromide. Images were captured with a camera and the relative band intensities were analyzed with a computer-assisted bioimaging analyzer (EXT-20MX; Vilber Lourmat) after normalization against actin.

### 2.6. Statistical Analysis

All data are presented as means ± SE. Multiple comparisons were performed using a one-way ANOVA with post hoc test using Tukey's method. IBM SPSS Statistics 20 (IBM) was used for analysis, and differences were considered statistically significant when *P* < 0.05.

## 3. Results

### 3.1. Assessment of Animal Model for Lymphedema Formation

**Figure 2:**
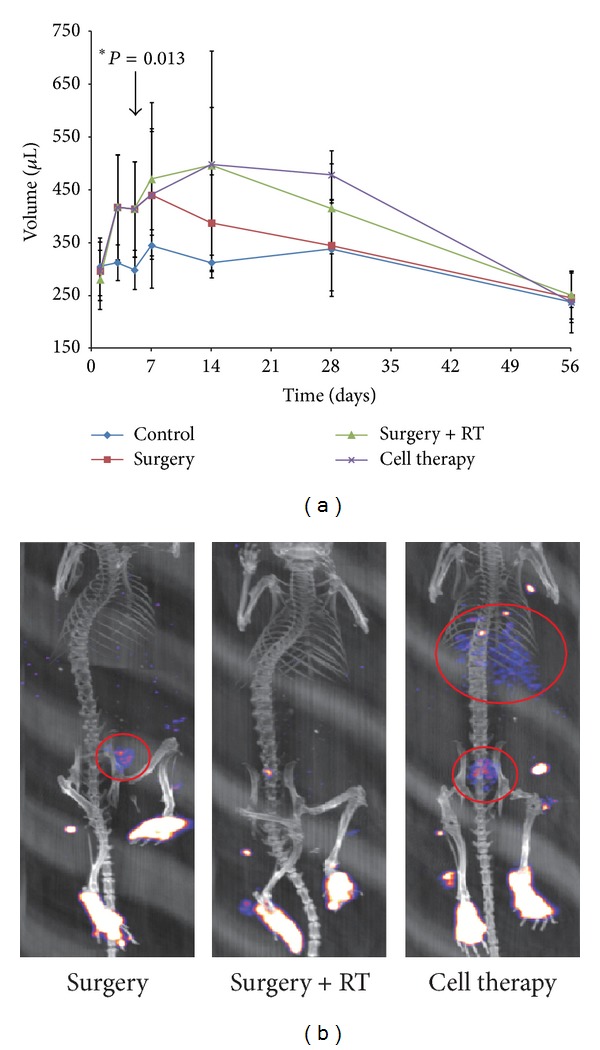
In vivo measurement of hindlimb volume and lymphatic flow. (a) Volume measurements of hindlimbs at different time intervals and compared against the normal left hindlimb. There is an overall increase in volume compared to the normal hindlimb, with statistical significance at day 5. This volume increase tends to be maintained after radiotherapy at 14 days, but this difference is lost at 56 days. (b) Lymphoscintigraphy shows an improvement in lymphatic flow after cell therapy as demonstrated by the collection of radioactive materials (red circles) in the proximal parts of the body compared to the other groups.

The degree of lymphedema in our animal model was measured by volumetric analysis using a water displacement method. The Surgery group showed a significant increase in volume of the operated right hindlimb compared to the contralateral normal hindlimb (used as control) at day 5, and there was a tendency for this difference to be maintained up to 1 week (Figure 2(a)). However, there was a decline in volume after 1 week, becoming similar to the normal contralateral hindlimb at 4 weeks, thus demonstrating that surgery alone was not able to produce a sustained effect for the study of chronic lymphedema. With the addition of radiotherapy at day 5, the Surgery+RT group showed a sustained volume increase after 1 week which was maintained up to 4 weeks compared to the Surgery group, although this difference failed to achieve statistical significance. Unfortunately, there was a high incidence of necrosis in hindlimbs of mice from the Surgery+RT group between 4 and 8 weeks, probably due to the toxicity of radiation exposure, which led to significant tissue loss, causing a dramatic decrease in volume during this period. 

Lymphoscintigraphic findings between the Surgery and Surgery+RT groups demonstrate that, in the Surgery group, there was partial radioactive uptake in the inguinal area in the delayed images which was not observed in the Surgery+RT group (Figure 2(b)). In the Surgery+RT group, there was barely any movement of radioactive substance from the foot. These results have two implications: (1) RT was probably able to ablate the remnant lymphatics that were not fully destroyed by surgical methods, and (2) RT was able to suppress spontaneous regeneration of lymphatic vessels, thus completely obstructing lymphatic flow leading to increased hindlimb volume.

### 3.2. MDSCs Cocultured with HLEC

**Figure 3:**
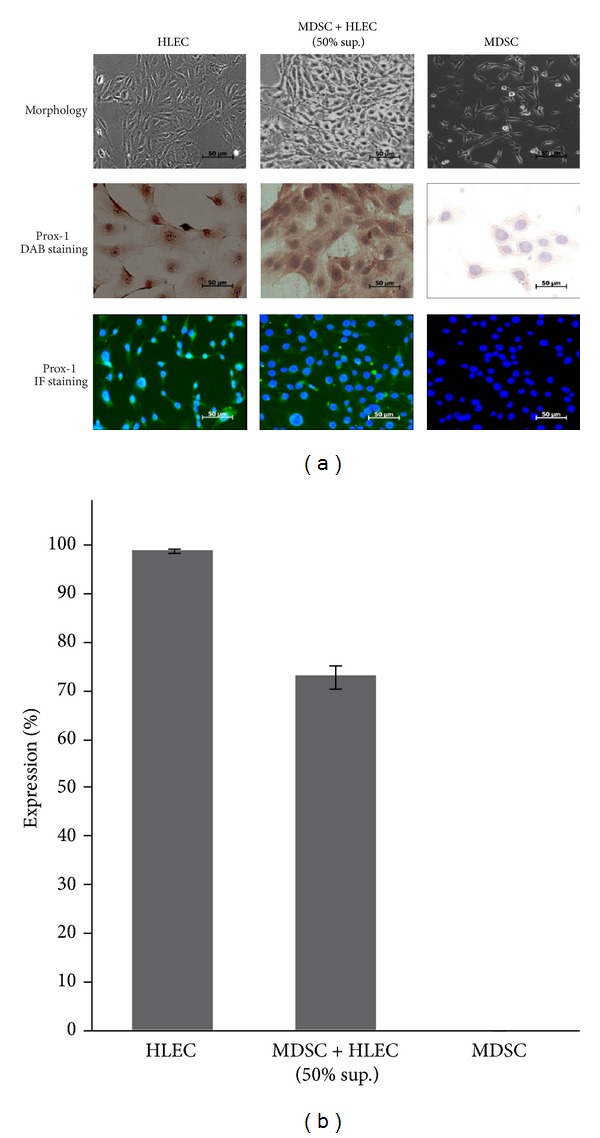
Coculture of MDSCs with the supernatant of HLECs demonstrates differentiation of MDSCs towards the lymphatic lineage. (a) The coculture MDSC + HLEC (50% sup.) shows a change in morphology, as shown by the change from a spindled shape (MDSC) to a rounder shape, resembling the morphology of HLECs. The coculture also shows positive expression of Prox-1 under DAB and IF staining, while native MDSCs show negative expression (400x magnification). (b) Graphical representation of Prox-1 expression rate showing 70% expression in the coculture compared to negative expression in native MDSCs.

**Figure 4:**
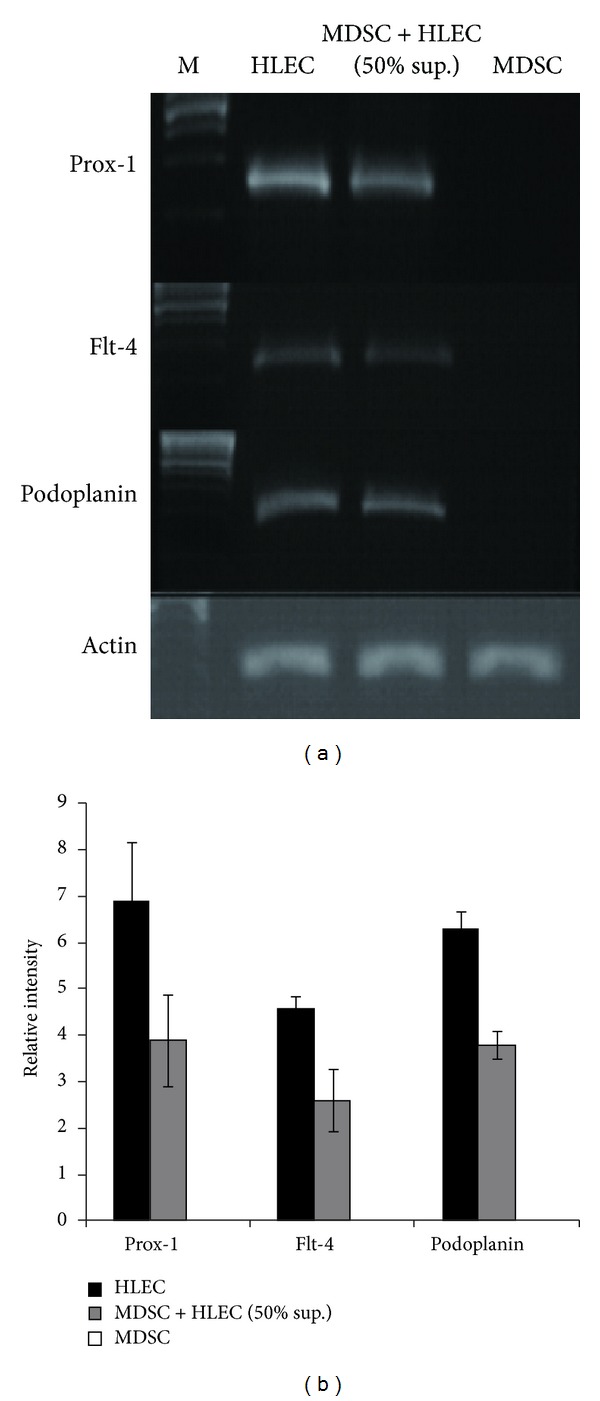
Upregulation of lymphatic specific genes after coculture of MDSCs with the supernatant of HLECs. (a) RT-PCR demonstrates negative expression of genes for Prox-1, FLt-4, and podoplanin in native MDSCs, while the coculture demonstrates upregulation of these genes. (b) Graphical representation of gene expression rate normalized against actin. There is upregulation of genes for Prox-1, FLt-4, and podoplanin after coculture compared to native MDSCs.

In order to assess the applicability of our animal model for studies of lymphangiogenesis, we differentiated stem cells into LEC precursors in vitro. MDSCs cocultured with the 50% supernatant of HLECs showed a change in cell morphology from a spindled shape to round shape under light microscopy, resembling the appearance of HLECs (Figure 3(a)). DAB staining of cells with lymphatic marker Prox-1 showed that native MDSCs had no expression, while cocultured MDSCs showed positive brown staining of the cytoplasm in morphologically MDSC-like cells (as shown by the same morphology of the nucleus). IF staining for Prox-1 also revealed a similar pattern, with MDSCs showing only DAPI stained nuclei while the coculture, as well as HLECs, showed a positive green fluorescence of the cytoplasm. Quantification of Prox-1 expression demonstrated that MDSCs had no expression while the coculture showed an expression rate of around 70% relative to HLECs (100%) (Figure 3(b)). At the genetic level, RT-PCR also showed a similar pattern, with native MDSCs showing no gene expression for lymphatic markers Prox-1, Flt-4, and podoplanin, while the coculture showed around 60% expression of these lymphatic markers with respect to HLECs (Figures 4(a) and 4(b)). These in vitro studies demonstrate that a coculture of MDSCs with the supernatant of HLECs can drive differentiation of MDSCs towards the lymphoendothelial lineage, showing expression of lymphatic markers at the genetic and cellular levels while maintaining the morphologic characteristics of MDSCs.

### 3.3. Effect of Cell Therapy on Lymphedema Attenuation

Injection of lymphoendothelial precursor cells derived from MDSCs into the hindlimbs after RT did not cause any attenuation of hindlimb volume during the whole study period (Figure 2(a)). Volumetric analysis of the Cell therapy group showed a similar pattern of volume to that of the Surgery+RT group, with a steep decline in volume after 4 weeks due to tissue loss. Lymphoscintigraphy, on the other hand, showed increased radioactive substances in the upper part of the body in the delayed frame images (Figure 2(b)), suggesting that lymphatic flow may be restored after cell therapy.

**Figure 5:**
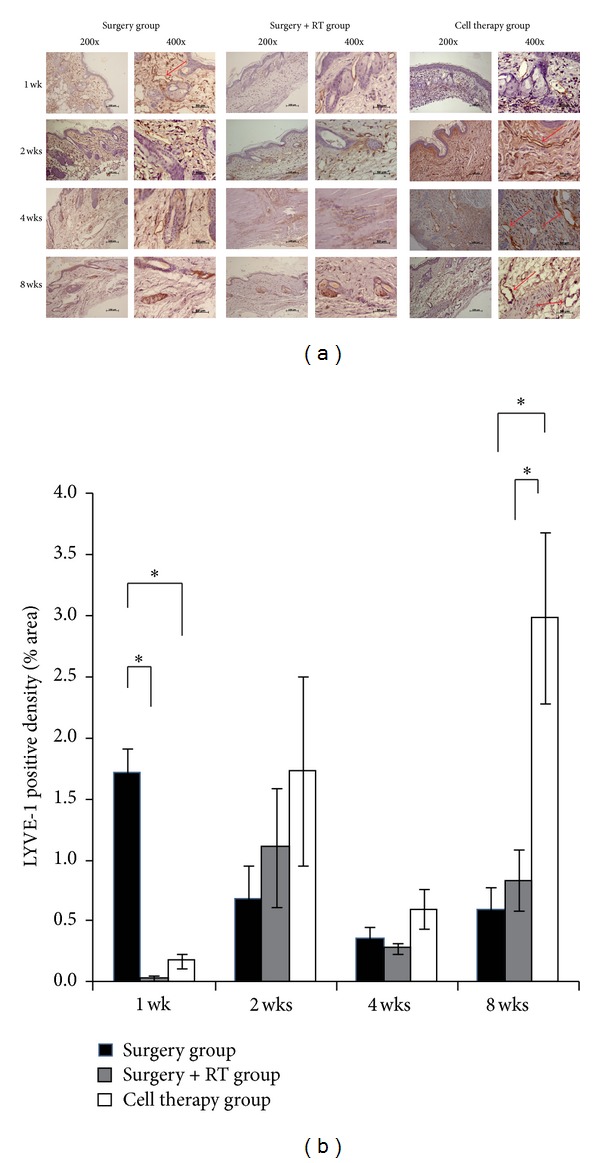
Increase in formation of LYVE-1 positive lymphatic structures after injection of differentiated stem cells (a) LYVE-1 IHC staining from harvested tissues at 1, 2, 4, and 8 weeks, showing increased formation of LYVE-1 positive lymphatic structures (arrows) in the Cell therapy group. (b) Graphical representation of LYVE-1 positive lymphatic structure density, showing an overall increased density at 2, 4, and 8 weeks in the Cell therapy group. Asterisk (∗) represents *P* < 0.05.

At the histological level, IHC for LYVE-1, a cell surface receptor on lymphatic endothelial cells, was performed from the specimens obtained at 1, 2, 4, and 8 weeks. The results showed that the Cell therapy group had an overall higher expression of brown stained tubular structures compared to the Surgery+RT or Surgery group (Figure 5(a)). Quantification of the lymphatic structures showed that at 1 week the Surgery+RT and Cell therapy groups had significantly lower number of lymphatics compared to the Surgery group (Figure 5(b)). This finding can be explained by the fact that radiotherapy and cell injection were done at day 5 and therefore there was immediate destruction of lymphatics by radiation without enough time for the effects of cell therapy to appear. However from 2 weeks, the Cell therapy group had higher lymphatic formation than the other groups which becomes statistically significant at 8 weeks. 

## 4. Discussion

In this study we attempted to create a modified, more radical animal model of chronic lymphedema. Our animal model was similar to the prototype model proposed by Olszewski et al. [10] in canine limbs, which was first modified by Wang and Zhong [11] to be performed in rodents. Wang's rat hindlimb model involved circumferential incision and excision of a circular strip of skin and all subcutaneous tissue, identification and ligation of the main lymphatic trunks after dye injection, popliteal node excision, and suturing of the skin edges to the muscles. Lymphedema persisted up to 15 days postoperatively, but the difference was lost by 30 days, and the difference became significant again after 180 days. Despite the late reappearance of lymphedema after 6 months, this model was only partially successful in producing a sustained effect needed to study chronic lymphedema. In our study, we performed a more radical surgical ablation of the lymphatics, especially the deep lymphatic system, which may not have been fully ablated in Wang's model. To achieve this, we also resected the muscle down to the periosteum to fully divide all underlying deep lymphatics, and, after meticulous electrocauterization of any stained lymphatics, the muscle was sutured again to its original location. We also performed electrocauterization around the upper and lower circumferential resection margins, and the inguinal lymph nodes were removed using microsurgical techniques. Despite this radical surgical approach, our results showed that the edematous state did not persist beyond 1 week. Addition of radiation therapy was therefore performed in an attempt to fully destroy any remnant lymphatics that may not have been destroyed under surgical methods and to suppress spontaneous lymphatic vascular regeneration that may occur after surgery. Both Lee-Donaldson et al. [12] and Kanter et al. [13] used radiotherapy to induce lymphedema, but, in these studies, the degree of surgical ablation of the lymphatics was less radical, ranging from regional groin lymphatic ablation with lymph node dissection only (Lee-Donaldson) to circumferential excision of skin and subcutaneous tissue and deep lymphatic ablation after dye injection but without muscle resection or lymph node dissection (Kanter). The radiation dose used in our study was 4500 rads, which was the same dose used by the previous studies on rodents. Lymphedema was sustained for a longer period of time after radiotherapy, but necrosis of the hindlimb after 4 weeks was found, probably due to the toxicity of radiation, making further volumetric analyses inappropriate. The same problems were also faced in the previous studies, suggesting that an adjustment of the radiation dose is necessary to overcome this problem. In fact, previous studies in canine models have used radiation doses of 1200 to 1500 rads [14, 15], suggesting that the dose of 4500 rads in smaller rodents may be too toxic. Yet, our results are in accordance with previous studies in that RT is a necessary procedure to induce sustained lymphedema after surgery. The fact that the radicality of the surgical procedure to destroy potential deep lymphatics did not affect the duration of lymphedema may suggest that the role of RT is greater in the suppression of lymphatic regeneration and collateral circulation rather than total ablation of remnant lymphatics.

In terms of the applicability of our model for the study of therapeutic lymphangiogenesis, the necrosis of the hindlimb tissue after RT made volumetric analysis studies inappropriate. A refinement of the model is necessary, yet it is also possible that the cell therapy effect may not have been enough to produce substantial changes in volume. One possible explanation may be that the therapeutic dose may not have been achieved by a single injection. A previous study of stem cell lymphangiogenesis achieved lymphedema regression by performing weekly injections of stem cells [16]. Despite these problems, the lymphoscintigraphic findings and the increased lymphatic vessel formation in our IHC studies after cell therapy suggest that the model can be used to analyze functional aspects of lymphatic flow and provide for histopathologic information, the latter being an advantage of the rodent hindlimb model compared to the tail model (abundant tissue for harvesting).

In our study, we did not investigate the histopathologic characteristics associated with chronic lymphedema, which include findings such as increased number of fibroblasts, adipocytes, keratinocytes, mononuclear inflammatory cells, and ultimately skin thickening with subcutaneous tissue fibrosis [17]. However, we found that calgranulin (both A and B) genetic expression was increased from RT-PCR studies of harvested hindlimb tissues at 4 and 8 weeks in the Surgery+RT group and even higher in Cell therapy group (data not shown). Calgranulin is a gene known to be related to inflammation and it has been reported to be expressed with high specificity in microarray studies of lymphedema [18]. RT has the effect of producing an inflammatory response which is needed to produce the effects observed in human chronic lymphedema, and a higher inflammatory response in the Cell therapy group can be explained by the fact that inflammatory response to lymph stasis acts as an important trigger for postnatal lymphangiogenesis [19, 20].

With regard to therapeutic lymphangiogenesis, we have proposed a different approach using stem cells instead of the more commonly used VEGF-C. Previous studies have mainly used VEGF-C administered either as single injections [21] or by gene transfer into plasmid DNAs encoding human VEGF-C [22]. Other growth factors such as HGF [23] and PDGF-BB [24] have also been reported. However, stem cells have the advantage that they have potential for multilineage differentiation and self-regeneration [25], which can be used to increase lymphangiogenesis effectively. The role of stem cells in lymphangiogenesis was first described by Liersch et al. [26] by use of embryonic stem cells stimulated with both VEGF-C and VEGF-A, but, since then, only few reports on the potential role of stem cells for treatment of lymphedema can be found [16, 27–29]. Our study is one of the few studies that have used adult somatic stem cells, with the advantage that MDSCs originate from one of the most abundant and easily obtainable sources, and its efficacy and freedom from malignant transformation have been widely studied [30].

Overall, we were not able to create a reliable model of chronic lymphedema despite our more radical approach using both surgical and radiotherapeutic methods. Our study was also limited by a short study period of only 8 weeks, and thus late effects such as delayed reappearance of lymphedema could not be assessed. The effects of cell therapy on attenuation of lymphedema were not evident from a volumetric approach, which demonstrates the need for a refined animal model, especially a titration for optimal radiation dosage. However, the tendency for functional improvement of lymphatic flow and increased lymphatic vessel formation suggest that there is still room for improvement of this model and that stem cells can be a promising tool for use in therapeutic lymphangiogenesis. 

## Figures and Tables

**Table 1:** RT-PCR primer sequences and product size.

Gene	Accession number	Primer sequence (forward, reverse)	Temp (°C)	Size (bp)
Prox-1	NM_002763.3	5′-GGAGATGGCTGAGAACAAGC-3′	53	232
		5′-AGACTTTGACCACCGTGTCC-3′		
Flt-4	NM_182925	5′-GCTGTTGGTTGGAGAGAAGC-3′	53	213
		5′-TGCTGGAGAGTTCTGTGTGG-3′		
Podoplanin	BC022812	5′-GCCAGTGTTGTTCTGGGTTT-3′	53	209
		5′-AGAGGTGCCTTGCCAGTAGA-3′		
Actin	NM_001101.3	5′-GAGTCAACGGATTTGGTCGT-3′	51	200
		5′-TTGATTTTGGAGGGATCTCG-3′		
